# Determinants of HIV related mortality in African children on antiretroviral therapy: clinical and immunological insights from a systematic review and meta-analysis

**DOI:** 10.1186/s12981-025-00784-y

**Published:** 2025-10-08

**Authors:** Sisay Moges, Bereket Aberham Lajore

**Affiliations:** Department of Family Health, Hossana College of Science, Hosanna, Ethiopia

**Keywords:** Predictor of mortality, HIV-related mortality, Children, Clinical and immunological factors, Africa, Systematic review, meta-analysis

## Abstract

**Background:**

The HIV epidemic in Africa is still a serious public health concern, particularly for children who are more vulnerable to its negative consequences. Various studies carried out in different African nations have shown associations between these variables and increased mortality risk in children receiving antiretroviral therapy. However, the magnitude and consistency of these effects across different settings in Africa remain unclear, with a few studies reporting nonsignificant effects of advanced disease stage and immunological factors on mortality. This review is the first to provide a thorough analysis of the determinants of HIV-related mortality in children.

**Methods:**

This review followed the PRISMA guidelines, and relevant studies were obtained from the PubMed, CINAHL, EMBASE, and Google Scholar databases. Study selection, data extraction, and quality evaluation were carried out separately by two reviewers. A heterogeneity-based meta-analysis was conducted using random effect models. A sub group analysis was done based on age group and country.

**Results:**

A total of 36 studies involving 198,957 study participants were included in the review. Advanced disease stage (WHO III/IV) (HR 3.45; 95% CI 2.17–5.48), TB coinfection (HR 2.12; 95% CI 1.53–2.92), opportunistic infections (HR 2.04; 95% CI 1.59–2.62), immunosuppression (HR 2.50; 95% CI 2.01–3.11), and poor medication adherence (HR 3.36; 95% CI 2.10–5.38) and lack of cotrimoxazole use (HR 2.2; 95% CI 1.14–4.26) were significantly associated with a greater risk of HIV-related mortality.

**Conclusion:**

This review revealed key clinical, immunological, and treatment-related predictors of HIV-related mortality in children in Africa, including advanced disease stage, TB co-infection, immunosuppression, poor adherence, and lack of cotrimoxazole use. To reduce HIV-related child mortality in Africa, health policies should strengthen pediatric HIV care through context-specific service delivery. This includes early identification of advanced disease, management of opportunistic infections, access to cotrimoxazole prophylaxis, and age-appropriate adherence support, especially in under-resourced settings.

**Supplementary Information:**

The online version contains supplementary material available at 10.1186/s12981-025-00784-y.

## Background

The HIV epidemic in Africa remains a major public health issue, especially affecting children who are more susceptible to its harmful effects [1]. The WHO estimates that by the end of 2022, 1.3 million children in the African area, aged 0 to 14, were living with HIV, and around 69,000 of them lost their lives to AIDS-related causes [2]. Severe immunological suppression at the time of ART beginning and advanced disease stage are the primary crucial variables that are consistently related to death of children with HIV [3, 4]. HIV progression is divided into four stages by the WHO clinical staging system, with stages III and IV denoting advanced disease characterized by severe opportunistic infections and diseases that define AIDS. Similarly, the CD4 + T-cell count or percentage, an important indicator of immune health, offers vital insights into the degree to which the immune system weakens in people living with HIV [5, 6]. Moreover, TB coinfection and opportunistic infections were some of the other significant predictors of child mortality with HIV infection [7–9]. Various studies carried out in different African nations have shown associations between these variables and increased mortality risk in children receiving antiretroviral therapy. For instance, studies [10–15] have shown that clinical factors such as late-stage disease are associated with a significantly greater risk of death. Similarly, studies [8, 10, 11, 14, 16] reported that severe immune suppression was a strong predictor of mortality in a cohort of HIV-infected children. However, the magnitude and consistency of these effects across different settings in Africa remain unclear, with a few studies reporting nonsignificant effects of advanced disease stage and immunological factors on mortality [15].

Furthermore, although a comprehensive review of the HIV mortality rate in Africa has been conducted [17], it primarily focused on pooled mortality estimates and did not explore the underlying risk factors contributing to child mortality among HIV-infected patients. This review is the first to provide a thorough analysis of the clinical and immunological determinants of HIV-related mortality in children. Therefore, a comprehensive review is warranted to determine the effects of clinical and immunological factors on HIV-related mortality. Additionally, this review adds to the larger argument on how to lower pediatric HIV mortality in areas with limited resources. Measuring the effect of immunological and clinical determinants on death would provide solid data for early diagnosis, care coordination, and the start of ART for children with HIV. By addressing important risk factors for HIV-related child mortality in Africa, this review aims to fill a critical knowledge gap and offer insightful guidance for future research, interventions, and policy decisions aimed at improving the survival and well-being of this vulnerable population.

The main aim of this review is to identify determinants of HIV related mortality in African children on antiretroviral therapy focusing on the clinical and immunological factors.

## Methods and materials

This review is reported in accordance with the guidelines established by the Preferred Reporting Items for Systematic Reviews and Meta-Analyses Protocols (PRISMA-P) statement [18].

### The eligibility criteria

*Population* (P) HIV-infected children aged 0–15 years receiving antiretroviral therapy (ART) in one or more African countries.

*Intervention/Exposure (I)* Advanced HIV disease stage (WHO clinical stage III or IV), TB co-infection, Opportunistic infection and immune suppression (defined by CD4 count or age-appropriate percentage thresholds).

*Comparison (C)* Children with less advanced disease stages (WHO clinical stage I or II) and without immune suppression.

*Outcome (O)* Mortality associated with specific exposures.

*Study Design/Eligibility Criteria* Retrospective cohort, case-control, and cross-sectional studies, as well as theses and dissertations published in English between 2010 and 2024. Studies were excluded if they involved single case reports, case series, reviews, comments, or editorials, lacked relevant outcome data, or were inaccessible after two attempts to contact the authors.

### Databases and search strategy

Several electronic databases, including PubMed, Embase, Hinari CINAHL, African Journals Online (AJOL), OpenGray, Google Scholar and Science Direct, were searched for peer-reviewed published publications. For searching literature the following keywords and phrases were used. HIV/AIDS; death OR survival; predictor OR associated factor OR risk factors; OR determinants; effect of immunosuppression; effect of severe disease stage; antiretroviral therapy OR ART; pediatrics OR children; under five years OR 15 years [African countries]. Methodological terminologies including cross-sectional, cohort, prospective, and retrospective studies were also used. Boolean operators (AND, OR) were used to combine these keywords to create long search strings that will locate relevant research across several databases. For example, the following search strategy was employed in the PubMed database as an example: (“HIV Infections” [MeSH] OR “HIV” [All Fields] OR “HIV/AIDS” [All Fields]) AND (“Mortality” [MeSH] OR “Survival” [All Fields] OR “death” [All Fields]) AND (“Children” [MeSH] OR “Child” [All Fields] OR “Pediatric” [All Fields]) OR “Under five years” [All Fields]) OR “under 15 years” [All Fields]) AND (“Antiretroviral Therapy, Highly Active” [MeSH] OR “ART” [All Fields] OR “Antiretroviral therapy” [All Fields]) AND (“Sub-Saharan Africa“[MeSH] OR “Africa“[All Fields]) AND (“Determinants” [All Fields] OR “Predictors” [All Fields] OR “Associated Factors” [All Fields] OR “Risk Factors” [All Fields]) OR “The effect of Advanced Disease” [All Field]. This search combined relevant MeSH terms and free-text words to capture studies on risk factors for HIV infections.

### Study screening and selection process

The study screening and selection followed a systematic approach. Two independent reviewers (SM & BAL) screened the titles and abstracts of all records retrieved from databases and manual searches, using predefined eligibility criteria. Duplicate entries were removed using EndNote XX. Full texts of potentially eligible studies were obtained and assessed independently to ensure they met the inclusion and exclusion criteria. Studies that failed to qualify were omitted and a reason would be given. Any differences involving the screening or selection process were decided through a discussion.

### Data extraction

Data extraction was conducted using a Microsoft Excel spreadsheet based on a predefined checklist. The checklist included details such as the first author’s name, publication year, study participants, study country, and sample size, types of determinant, the HR and its 95%CI. To ensure consistency and accuracy, both reviewers initially extracted data independently and resolved any discrepancies through discussion.

### Quality assessment

Two independent reviewers carefully assessed the risk of bias using the Newcastle–Ottawa Scale (NOS) for observational studies [19]. This tool evaluates bias across three key domains: study group selection, group comparability, and outcome assessment. The NOS assigns up to four stars for selection, two for comparability, and three for outcome evaluation, providing a comprehensive quality assessment. The findings are summarized in the ‘Summary of Findings’ table.

### Variable measurement and definition

The primary outcomes of interest in this review were advanced disease stage (late stage of HIV disease progression), described using WHO clinical staging and WHO Stage III and IV were considered as advanced disease stage. Another outcome is immunosuppression, defined as a CD4 count below a specific threshold. Most of the included studies [10, 13, 20–23] classified immunosuppression as follows: CD4 cell counts < 1500/mm³ or < 25% for patients aged < 12 months, CD4 cell counts < 750/mm³ or < 20% for patients aged 12–35 months, and CD4 cell counts < 350/mm³ or < 15% for patients aged 36–59 months. One study used the Nadir immunosuppression classification, categorizing it as severe (CD4% < 15% or count < 200 cells/µl) or moderate (CD4% of 15–24% or count of 200–350 cells/µl) [11]. Another study defined immunosuppression as a CD4 count < 350 cells/mm³ [15]. Poor adherence was defined as adherence below 85%, indicated by missing 6 doses out of 30 or 10 or more doses out of 60, as documented by the ART physician [14, 24, 25]. Other outcomes of interest included TB coinfection, opportunistic infections (other than TB), and cotrimoxazole nonuse. A random-effects model was employed to pool the mortality risk specific to each outcome across studies, accounting for potential heterogeneity.

### Data synthesis and analysis

A narrative synthesis was used to describe the characteristics of the included studies, while a meta-analysis was carried out using Stata version 17. Hazard ratios (HRs) along with 95% confidence intervals (CIs) were computed, with HRs log-transformed to derive effect sizes and their corresponding standard errors. Forest plots were utilized to display the combined effect estimates. To assess statistical heterogeneity, Cochran’s Q test, the I² statistic, and the chi-square test (with significance set at *p* < 0.05) were applied. I^2^ statistic of 25%, 50%, and 75% represent low, moderate, and high heterogeneity respectively [26, 27]. Subgroup analyses were performed based on the country where the study was conducted and the age group of participants. Given the variability among studies, a random-effects model was employed to generate pooled effect estimates. Publication bias assessment was done using Egger’s regression-based test. The report was presented using texts, tables and plots.

## Results and discussion

### Study selection

**Fig. 1:**
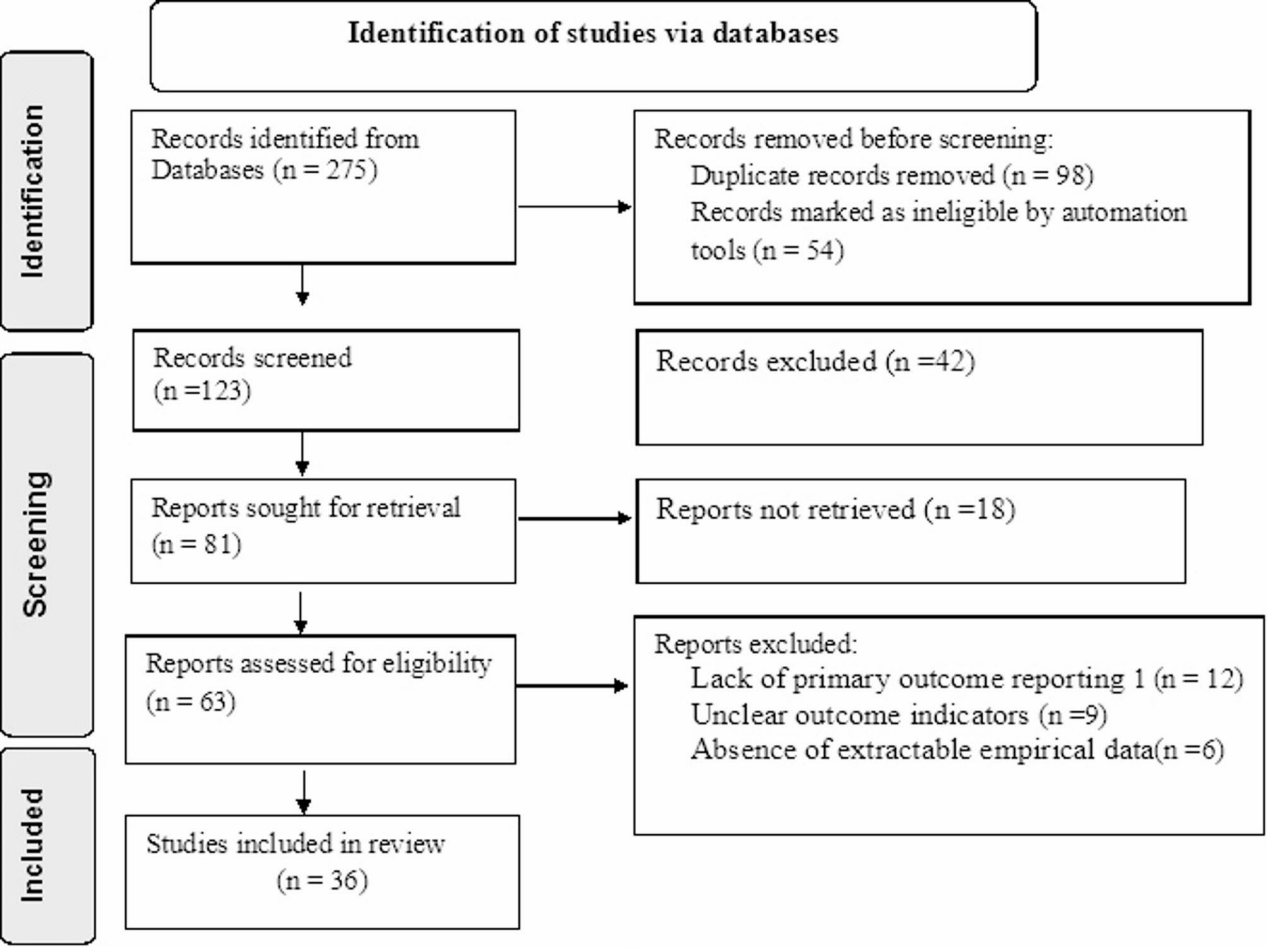
PRISMA flow diagram of the selection of studies among HIV infected children in Africa

A search across multiple scholarly databases produced 275 publications as indicated elsewhere [28]. There were several refining steps in the selection process, which was led by a PRISMA flow diagram [18]. Following a title assessment and duplicate removal, 152 items were eliminated. After a closer examination of the remaining 81 abstracts, 42 more were eliminated because they did not fit the inclusion requirements. The authors then evaluated the remaining 63 articles in full text. This thorough evaluation resulted in 36 articles meeting all eligibility requirements. The 27 excluded studies were omitted due to a lack of primary outcome reporting (12 studies), unclear outcome indicators (9 studies), or the absence of extractable empirical data (6 studies). In conclusion, this review included 36 studies examining how clinical and immunological factors influence HIV-related mortality in children (Fig. 1).

### Characteristics of the studies

The review included 36 studies that were conducted in African countries between 2010 and 2024 involving a total of 198,957 patients. All studies employed a retrospective cohort design. This review analyzed multiple clinical and immunologic factors affecting HIV mortality: the effect of advanced disease stage (WHO stage III/IV) was examined in 30 studies; 11 studies investigated the impact of TB coinfection; eight 8 studies assessed the effect of opportunistic infections (OIs); 22 studies evaluated the influence of immunosuppression (CD4 count below specified thresholds); 13 studies examined the impact of poor adherence to treatment; and 5 studies investigated the effect of cotrimoxazole nonuse. Eighteen studies [7, 10, 13, 14, 20, 22–25, 29–38] included from, two studies from Malawi [39, 40], two from Kenya [9, 15], two from Zambia [41, 42], and two from South Africa [16, 43]. Additionally, several multi-country studies were included: one encompassing Malawi, Uganda, and Kenya [11]; another covering Malawi, South Africa, Zambia, and Zimbabwe [12]; and one covering Malawi, Lesotho, and Swaziland [44]. Single-country studies were conducted in Cote d’Ivoire [45], Cameroon [46], Tanzania [8], the Democratic Republic of Congo (DRC) [47], Nigeria [48], and Zimbabwe [49] (Table 1).

**Table 1:** Characteristics of the studies included in the review, among HIV infected children in Africa

Authors	Study participants	Country	Sample size
Munthali et al., [41]	< 15 years	Zambia	65,448
Chekole et al., [31]	< 15 years	Ethiopia	588
Anigilaje & Aderibigbe, [48]	< 15 years	Nigeria	368
Bitew et al., [25]	< 15 years	Ethiopia	228
Mekonnen et al., [34]	< 5 years	Ethiopia	415
Alebel et al., [10]	< 15 years	Ethiopia	553
Fetzer et al., [40]	< 15 years	Malawi	258
Ebissa et al., [32]	< 5 years	Ethiopia	556
Tagesse & Abebe, [23]	< 15 years	Ethiopia	410
Edessa et al., [7]	< 15 years	Ethiopia	315
Adem et al., [29]	< 15 years	Ethiopia	560
Abrams et al., [43]	< 5 years	South African	272
Mulugeta et al., [13]	< 15 years	Ethiopia	757
Koye et al., [22]	< 5 years	Ethiopia	549
Auld et al., [45]	< 15 years	Cote d’Ivoire’	2110
Ben-Farhat et al., [11]	< 15 years	Malawi, Uganda and Kenya	3949
Brophy et al., [39]	< 15 years	Malawi	2203
Sidamo et al., [14]	< 15 years	Ethiopia	421
Marie et al., [33]	< 5 years	Ethiopia	376
Davies et al., [12]	< 15 years	Malawi, South Africa, Zambia and Zimbabwe	12,655
McHugh et al., [49]	< 15 years	Zimbabwe	385
Biyazin et al., [20]	< 15 years	Ethiopia	251
Nlend & Loussikila, [46]	< 15 years	Cameroon	221
Nyandiko et al., [9]	< 15 years	Kenya	6234
Gebremedhin et al., [21]	< 15 years	Ethiopia	432
Arage et al., [38]	< 5 years	Ethiopia	426
Mwiru et al., [8]	< 15 years	Tanzania	3144
Zanoni et al., [16]	< 15 years	South Africa	537
Andargie & Asmleash, [50]	< 15 years	Ethiopia	269
Melaku et al., [35]	< 15 years	Ethiopia	11,695
Kabue et al., [44]	< 15 years	Malawi, Lesotho, & Swaziland	2306
Oumer et al., [37]	< 15 years	Ethiopia	243
Molla et al., [36]	< 15 years	Ethiopia	721
Mutanga et al., [42]	< 15 years	Zambia	1039
Nugent et al., [47]	< 15 years	DRC	1010
Alemu et al., [24]	< 5 years	Ethiopia	415

### Quality assessment and risk of bias

We assessed the risk of bias using the Newcastle‒Ottawa Scale (NOS) for observational studies [19], which was evaluated by two independent reviewers. The NOS evaluates studies based on three domains: selection (maximum 4 stars), comparability (maximum 2 stars), and outcome (maximum 3 stars), with a total possible score of 9 stars. The study by Munthali et al., [41] received the highest score of 9 stars, indicating high quality. In contrast, Ebissa et al., [32] and Chekole et al., [31] received the lowest scores of 6 stars each, indicating moderate quality. This was primarily due to possible selection bias, which could limit how well their study populations represent the broader target groups. Regarding the selection domain, most studies [9, 12, 13, 16, 20–22, 24, 25, 29, 33, 34, 40, 41, 44–48, 50] scored 3 out of 4 stars, indicating generally good representativeness and selection procedures. However, some studies, such as Ebissa et al., [32], scored lower (2 stars) due to their potential selection bias. For comparability, almost all studies received a maximum of 2 stars, indicating adequate control for confounding factors. This suggests that most researchers account for important variables that could influence outcomes. However, the five studies [13, 31, 32, 42] received one star, indicating a potential influence of unaddressed confounding factors that may have impacted the validity of their findings. In the outcome domain, scores ranged from 2 to 3 stars. Studies scoring 3 stars, such as Koye et al., 2012 [22] and Alebel et al., [10], likely had more robust outcome assessment methods, longer follow-up periods, and better cohort retention; however, some studies [8, 13, 14, 21, 23, 25, 32, 33, 38] received two stars, indicating that there is inconsistency in measuring outcomes, especially for anemia. Overall, the majority of studies (33 out of 36) were classified as high quality, scoring 7 or more stars (Supplementary file).

### The effect of late-stage HIV disease progression on HIV-related mortality

**Fig. 2:**
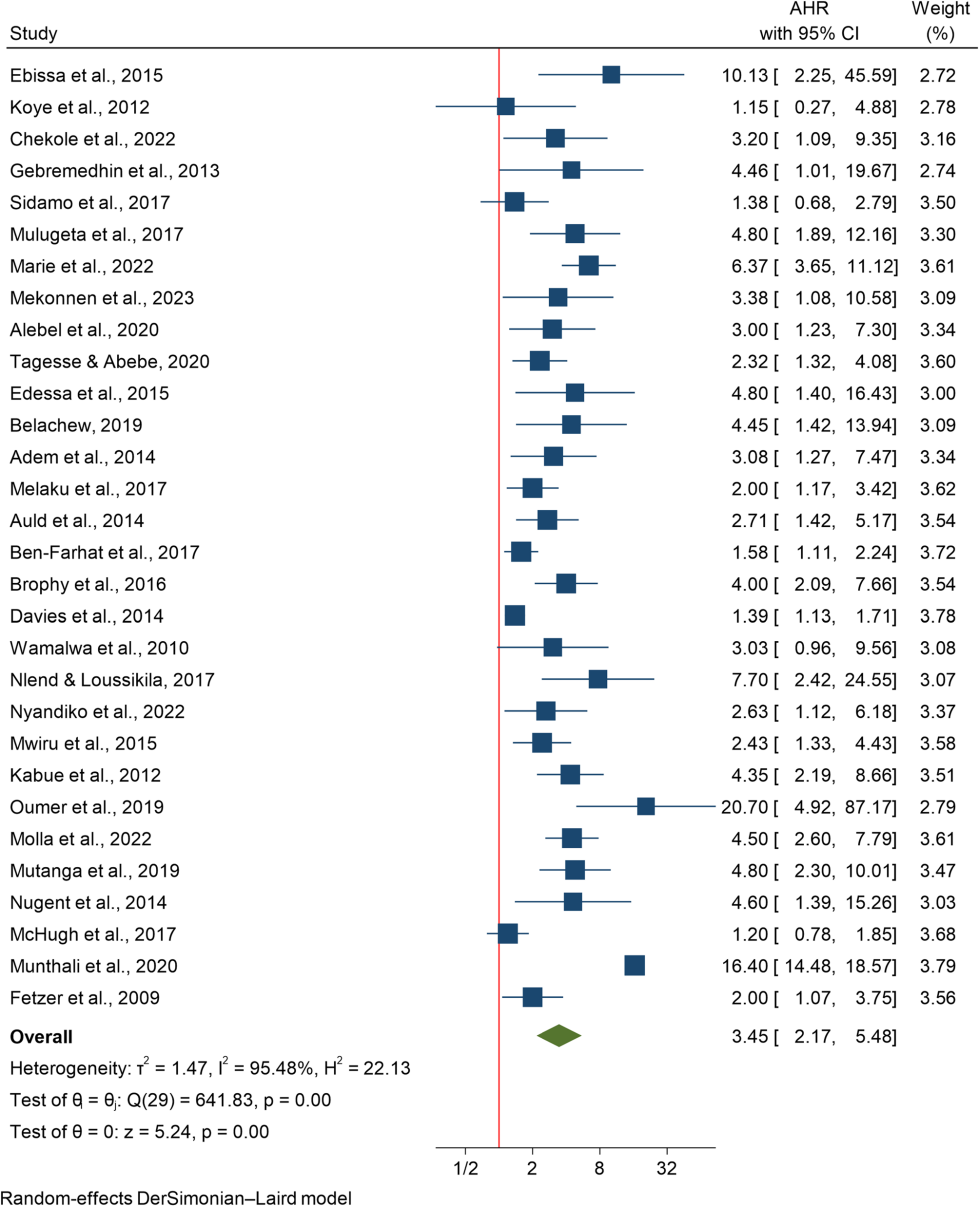
The association between WHO Stage III/IV and mortality among HIV infected children in Africa

The association between advanced disease progression and HIV mortality was assessed in 30 studies involving 113,775 participants. The mortality risk for patients in WHO clinical stages III/IV was 3.45 times greater than that for patients in stages I/II (HR 3.45; 95% CI 2.17–5.48) (Fig. 2).

### Subgroup analysis

Subgroup analysis revealed varying risks across different countries. Studies from Zambia [41, 42] reported the highest risk of mortality due to WHO stage III/IV (HR 9.379; 95% CI 2.827–31.114), followed by Cameroon [46] (HR 7.700; 95% CI 2.415–24.547) and Ethiopia [7, 10, 13, 14, 20, 22–25, 29–38] (HR: 3.490; 95% CI 2.562–4.754). In contrast, the lowest risk of mortality due to advanced disease was reported in a multi-country study covering Malawi, South Africa, Zambia, and Zimbabwe [12] (HR 1.390; 95% CI 1.130–1.710). A study from Zimbabwe [49] reported a non-significant result (HR 1.200; 95% CI 0.779–1.849). Furthermore, subgroup analysis done by age group indicated almost identical effect size among studies focusing on children below 5 years old (HR 3.461; 95% CI 1.690–7.088) [22, 32–35] and those including children up to 15 years of age (HR 3.453; 95% CI 2.048–5.824).

### Publication bias assessment

Publication bias was assessed using Egger’s regression-based test. The test yielded a beta coefficient of 0.87 (SE = 0.75), with a z-value of 1.16 and a p-value of 0.2473. Since the p-value is > 0.05, there is no statistically significant evidence of small-study effects or publication bias.

### The effect of TB coinfection on HIV-related mortality

**Fig. 3:**
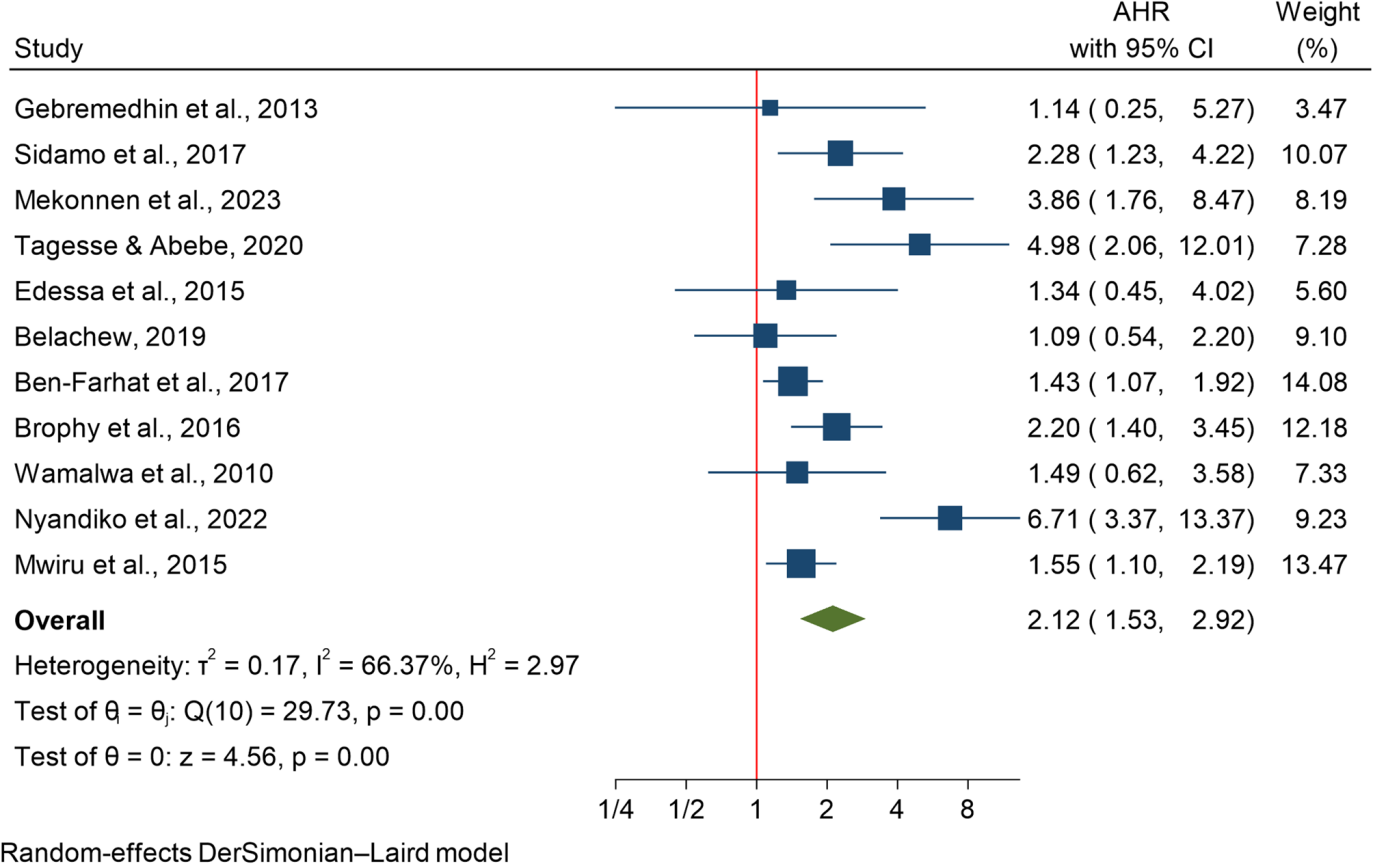
The association between TB co-infection and mortality among HIV infected children in Africa

Eleven studies encompassing 18,078 participants were analyzed the association between TB coinfection and mortality among HIV-infected children. Due to heterogeneity between studies (I²=66.37%), a random-effects meta-analysis was employed to pool the effect sizes. The results indicated that TB coinfection was associated with a twofold increase in mortality (HR 2.12; 95% CI 1.53–2.92) (Fig. 3).

### Subgroup analysis

Sub-group analysis indicated that the highest hazard of death due to TB co-infection was reported in a study from Malawi [39] (HR 2.200; 95% CI 1.403–3.449), followed closely by studies from Ethiopia (HR 2.168; 95% CI 1.295–3.629). In contrast, studies from Kenya [9, 15] reported a non-significant effect size (HR 3.243; 95% CI 0.743–14.158). Sub group analysis by age group indicated the risk of mortality due to TB coinfection varied by age group. Among children less than 5 years old, the risk was considerably greater (HR 3.860; 95% CI 1.760–8.468) [34] than that in the broader age group of children under 15 years (HR 2.003; 95% CI 1.442–2.784). This difference suggests that younger children with HIV and TB coinfection may be particularly vulnerable, highlighting the need for targeted interventions in this age group.

### Publication bias assessment

Publication bias assessment was done using Egger’s regression-based test for association between TB coinfection and HIV-related mortality. The test showed a beta coefficient of 0.26 (SE = 1.234), with a z-value of 0.21 and a p-value of 0.8309. Since the p-value is well above the 0.05 threshold, there is no statistically significant evidence of small-study effects or publication bias indicating the findings are likely not influenced by selective publication.

### Effect of opportunistic infection on HIV-related mortality

**Fig. 4:**
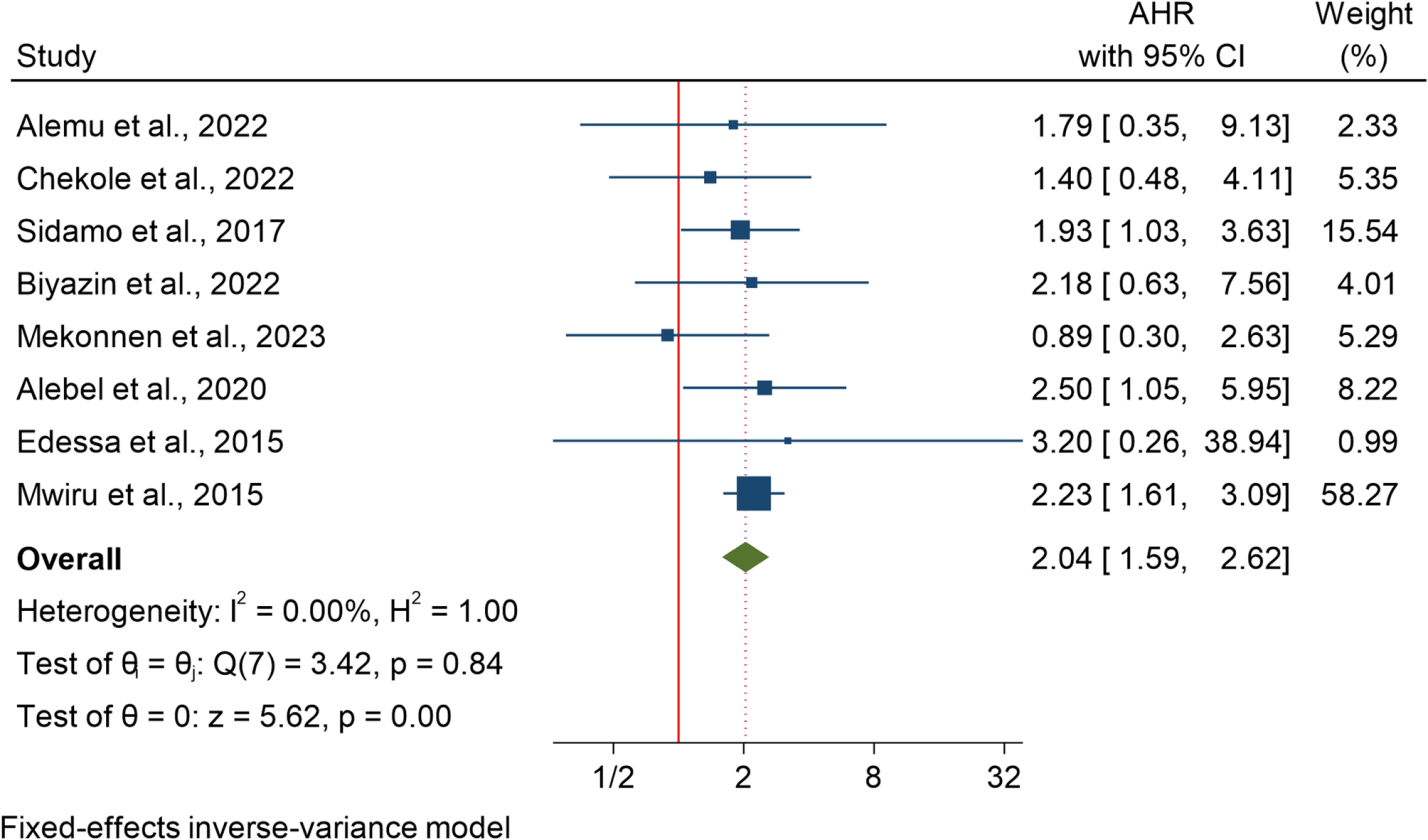
The association between opportunistic infection and mortality among HIV infected children in Africa

Eight studies encompassing 6,102 participants were analyzed to assess the association between opportunistic infections (OIs) and mortality using a fixed effects model. The results indicated that the presence of an OI was associated with a twofold increase in mortality (HR 2.04; 95% CI 1.59–2.62) (Fig. 4).

### Subgroup analysis

The subgroup analysis by age revealed that opportunistic infections were not significantly associated with mortality in children under 5 years (HR = 0.097; 95% CI − 0.804 to 0.998), while a stronger, borderline significant association was observed in children under 15 years (HR = 0.764; 95% CI 0.505 to 1.023).

### Publication bias assessment

A publication bias for association between opportunistic infection and HIV-related mortality was evaluated using egger’s regression-based test that yielded a beta coefficient of − 0.44 (SE = 0.640), with a z-value of − 0.68 and a p-value of 0.4948. Since the p-value > 0.05, there is no statistically significant evidence of publication bias.

### The effect of immunosuppression on HIV-related mortality

**Fig. 5:**
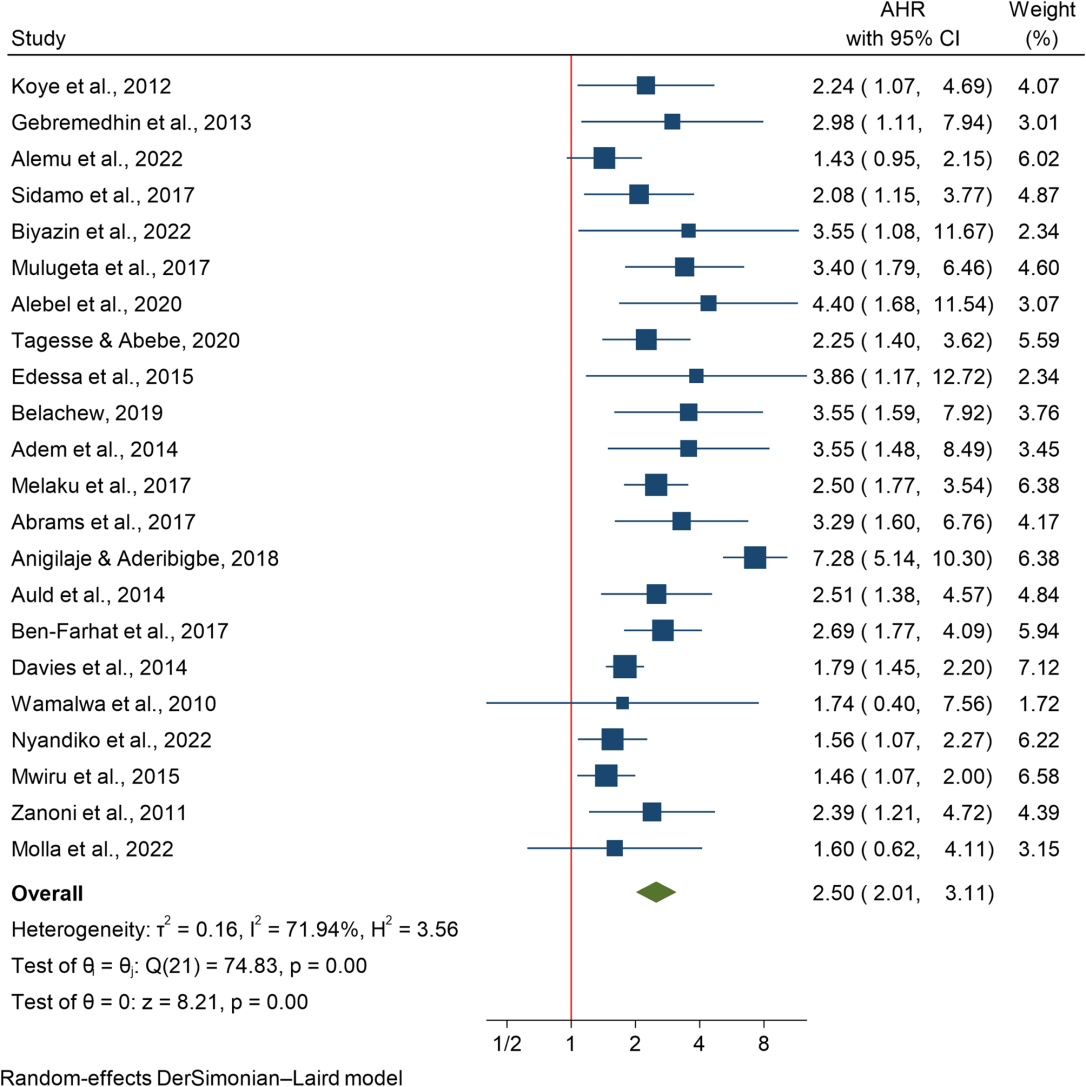
The association between immunosuppression and mortality among HIV infected children in Africa

The association between immunosuppression and HIV mortality was assessed in 22 studies involving 46,903 participants. Due to significant heterogeneity among studies (I²=71.94%), a random-effects model was employed to pool the risk estimates. Compared with those with CD4 counts above the thresholds, children with CD4 counts below the specified thresholds were associated with a 2.5-fold greater mortality risk (HR 2.50; 95% CI 2.01–3.11) (Fig. 5).

### Subgroup analysis

Subgroup analysis revealed geographical variations in the mortality risk due to immunosuppression. The highest risk was reported in a study from Nigeria [48] (HR 7.280; 95% CI 5.144–10.304), followed by South Africa [43] (HR 3.290; 95% CI 1.601–6.763). Studies from Ethiopia (HR 2.378; 95% CI 1.979–2.858) showed a pooled risk (HR 2.378; 95% CI 1.979–2.858). The lowest risk was reported in a study from Tanzania [8] (HR 1.460; 95% CI 1.068–1.996). Based on subgroup analysis by age group under 5 years and under 15 years old children, the hazard of death from immunosuppression varied by age group. Children under 15 years of age had a greater risk (HR 2.585; 95% CI 2.029–3.293) than did children less than 5 years (HR 2.039; 95% CI 1.222–3.404).

### Publication bias assessment

Egger’s regression-based test was applied to evaluate small-study effects for the association between immunosuppression and HIV-related mortality in children. The test produced a beta coefficient of 0.79 (SE = 0.731), with a z-value of 1.08 and a p-value of 0.2780. As the p-value > 0.05, there is no statistically significant evidence of publication bias, indicating the results are unlikely to be influenced by small-study effects or selective reporting.

### Effect of nonuse of Cotrimoxazole on HIV-related mortality

**Fig. 6:**
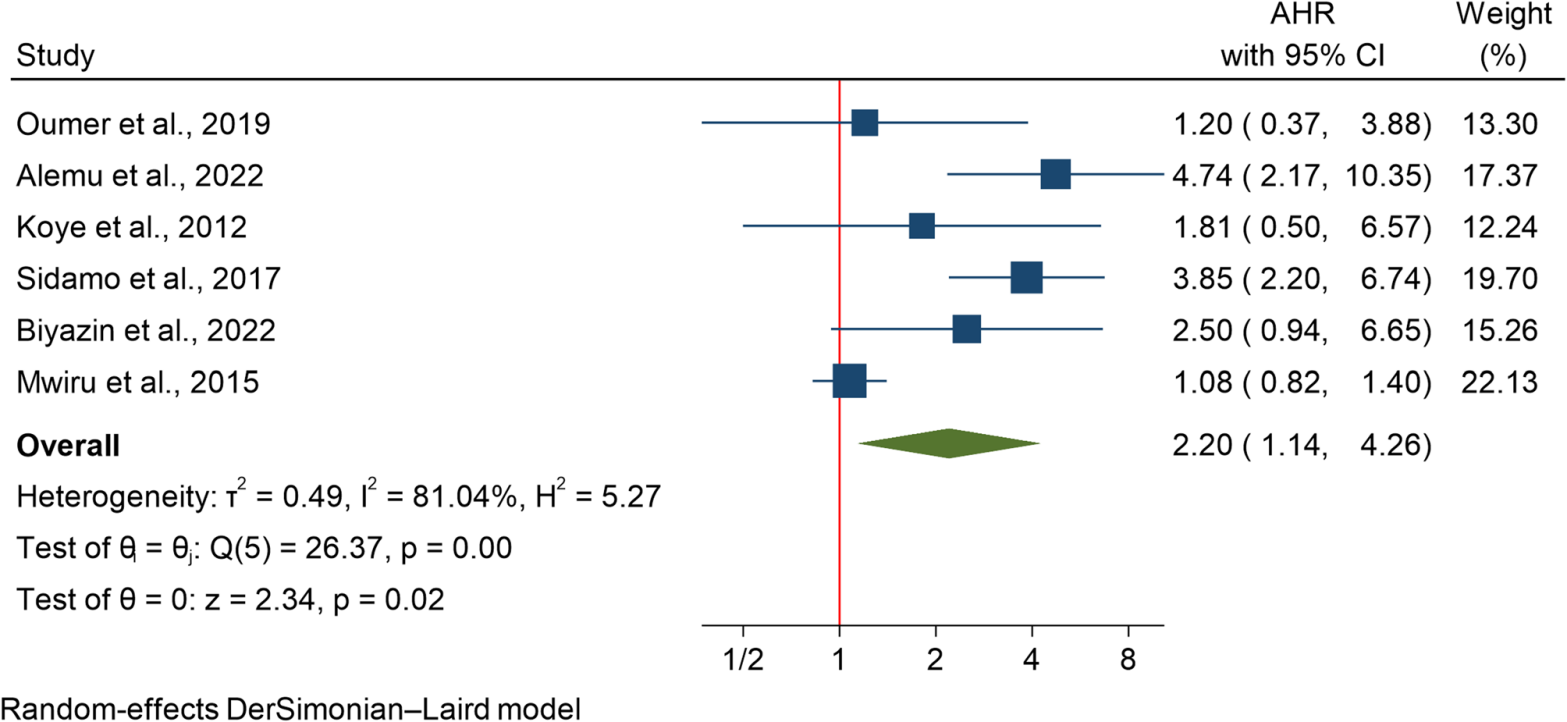
The association between none use of Cotrimoxazole and mortality among HIV infected children in Africa

The impact of cotrimoxazole none use on mortality risk was evaluated in 6 studies involving 5,023 participants. A random-effects model was employed due to significant heterogeneity among studies (I²=81.04%). The analysis revealed that individuals who did not receive cotrimoxazole therapy had twice the mortality than those receiving treatment (HR 2.20; 95% CI 1.14–4.26) (Fig. 6).

### Subgroup analysis

Subgroup analysis revealed the highest mortality rate due to cotrimoxazole nonuse was reported in Ethiopian studies (HR 3.019; 95% CI 1.945–4.686). In contrast, a study from Tanzania [8] reported a nonsignificant effect (HR 1.075; 95% CI 0.824–1.404). Moreover, based age based sub group analysis, there was age-related differences in mortality due to cotrimoxazole nonuse. Under 5 years of age exhibited a greater risk (HR 3.376; 95% CI 1.371–8.316) than did children under 15 years (HR 1.883; 95% CI 0.866–4.091). This finding suggests that younger children may be particularly vulnerable to the absence of cotrimoxazole therapy.

### Publication bias assessment

Egger’s regression-based test was conducted to assess small-study effects for the association between lack of cotrimoxazole use and HIV-related mortality in children. The test produced a beta coefficient of 0.35 (SE = 1.709), with a z-value of 0.21 and a p-value of 0.8371. As the p-value > 0.05 indicating that the results are unlikely to be affected by small-study effects or selective reporting.

### The effect of poor adherence on HIV-related mortality

**Fig. 7:**
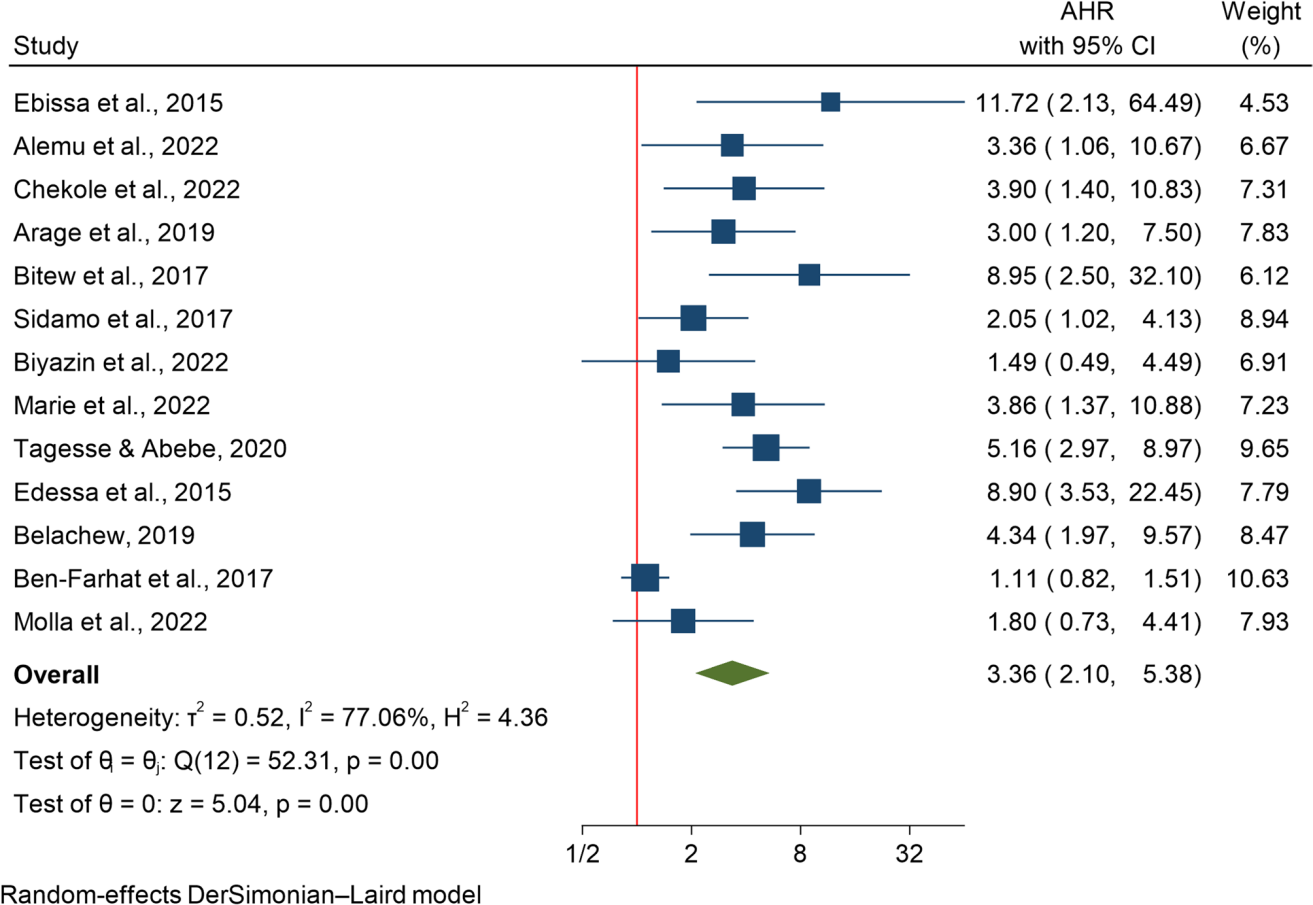
The association between poor adherence and mortality among HIV infected children in Africa

To evaluate the association between poor adherence and HIV mortality, thirteen studies including 9,076 participants were analyzed. A random-effects model was used due to heterogeneity (I² = 77.01%). The hazard of mortality in children with poor drug adherence was 3.36 times greater than that in children with better drug adherence (HR 3.36; 95% CI 2.10–5.38) (Fig. 7).

### Sub-group analysis

Subgroup analysis revealed that the highest death due to poor adherence was reported in Ethiopian studies (HR 3.751: 2.692–5.226), while a nonsignificant effect was reported in studies conducted in Malawi, Uganda, and Kenya [11] (HR 1.110; 95% CI 0.818–1.506). Moreover, based on subgroup analysis by age group, mortality due to poor adherence was greater among children less than 5 years old (HR 3.83; 95% CI 2.19–6.70) than among children under 15 years old (HR 3.13; 95% CI 1.74–5.61).

### Publication bias assessment

Egger’s regression-based test was performed to assess small-study effects for the association between poor medication adherence and HIV-related mortality in children. The test yielded a beta coefficient of 2.19 (SE = 1.393), with a z-value of 1.57 and a p-value of 0.1166. Since the p-value > 0.05, there is no statistically significant evidence of publication bias.

## Discussion

A comprehensive review of 36 studies conducted in different African countries involving 198,957 patients aimed to analyze the impact of clinical and immunological factors on HIV-related death among children in Africa. Significant predictors included advanced disease stage, TB coinfection and opportunistic infection, immunosuppression, poor adherence, and nonuse of cotrimoxazole. The current analysis revealed that advanced illness (WHO III/IV) substantially increased the probability of death by more than three times. This strong association between advanced disease stages and mortality is consistent with findings from China [51] that reported that children with advanced disease stages had significantly higher mortality rates. Nonetheless, compared to the impact size obtained from the pooled effect size among adults in Ethiopia, the risk of mortality owing to advanced illness stage in the current study was much greater [52]. The increased effect size in children implies that the influence of advanced illness stage on pediatric mortality can be considerably more significant [5]. This highlights how important it is that children receive an early HIV diagnosis and begin treatment.

TB coinfection doubles the risk of mortality, with a greater risk in children below 5 years of age than in older children. This finding aligns with global data on HIV/TB coinfection, which remains a leading cause of mortality in HIV-positive individuals [53, 54]. A report from review of adult studies in sub-Saharan Africa revealed that TB coinfection increased mortality risk by 1.8 times, which was slightly lower than the effect observed in children [55]. This could be because TB infection can affect the immunity of children, which can lead to mortality [56]. Moreover, the current review revealed, Ethiopia had highest effect size of TB co-infection and the risk of TB coinfection mortality was higher in younger children highlights the need for aggressive TB screening and preventive therapy in pediatric HIV care in sub-Saharan African countries and further investigation may indicate why the risk is highest in Ethiopia. In addition, the present analysis revealed that the risk doubled due to opportunistic infections. This result aligns with research conducted globally in both adult and pediatric populations [57, 58]. According to major US cohort research, opportunistic infections double the mortality risk of HIV-positive people [3]. This highlights the significance of timely treatment, prevention where necessary, and thorough screening for opportunistic infections.

The current analysis showed that immunosuppression (CD4 count under the threshold) increased the hazard of mortality in children infected with HIV. Consistent findings were reported in studies performed in Asia [59], in 17 middle- and high-income countries in Europe and Thailand [60], and in another study from Europe [61], which revealed that low CD4 + counts were significant predictors of mortality in children with HIV. The current review indicated that Nigeria had highest effect size of mortality due to due to severe immunosuppression. A meta-analysis in low- and middle-income countries revealed that severe immunosuppression (CD4 count < 50 cells/µL) increased mortality risk by 2.3 times, which is very similar to the effect observed in children [4]. This emphasizes how crucial it is to start ART early and perform routine CD4 monitoring to avoid developing severe immunosuppression. Physicians should address any conditions that might impair immunological recovery and prioritize immune reconstitution by efficient ART.

Poor medication adherence tripled the risk of death, particularly in children below 5 years of age. This strong effect of adherence on mortality is consistent with global data in both pediatric and adult populations, as evidenced by a report from Asia and Europe [60]. Moreover, research from Thailand [59] identified adherence as a major predictor of mortality in children living with HIV. This may be because noncompliance with treatment regimens is associated with immune impairment, extended viral replication, an increased risk of developing resistance to antiretroviral drugs, rapid progression of a child’s clinical condition, and a decreased likelihood of survival [6, 62]. However, the effect size in this report was greater than that found in a meta-analysis of adult studies [63] and the aforementioned report from Thailand [59]. The more pronounced effect size in children, especially younger ones, underscores the unique challenges associated with pediatric ART adherence [62]. Therefore, medication administration, guidance, and follow-up require critical supervision among children with HIV.

Consistent with previous reports [64, 65], the current analysis revealed that cotrimoxazole significantly reduced mortality in children with HIV. Not using cotrimoxazole increased the risk of death, with younger children at greater risk than older children. This finding is consistent with a double-blinded randomized controlled trial conducted among HIV-infected Zambian children receiving ART [66] and another study performed among infants in Africa [67]. One potential defense against serious infections such as cerebral toxoplasmosis, P. jirovecii pneumonia, bacterial pneumonia, and severe sepsis, all of which are significant contributors to illness and death in HIV-positive individuals, is the effectiveness of cotrimoxazole. These findings emphasize the important function of cotrimoxazole prophylaxis in treating HIV in children. It is essential to develop plans to increase the use of cotrimoxazole and ensure its reliable availability for treating HIV-positive children [68, 69]. These results highlight the critical role that cotrimoxazole prophylaxis plays in HIV therapy for pediatric patients. Strategies for improving the uptake of cotrimoxazole and guaranteeing consistent access are crucial in the treatment of HIV-positive children.

### Limitation

This review has several limitations. All included studies were observational, which restricts causal inference and introduces potential biases such as confounding and selection bias. Although formal tests showed no significant publication bias, the possibility of small-study effects cannot be entirely excluded. Notable heterogeneity was observed in some pooled estimates, likely due to differences regional variations and the pooled effect size should be interpreted cautiously. Most of the included studies were from Ethiopia, with limited representation from West and Central Africa, highlighting regional gaps and limiting the generalizability of findings across the continent. This also prevented subgroup analysis by country for certain factors.

## Conclusion

This systematic review and meta-analysis, which included 36 studies with 198,957 patients in Africa, provides strong evidence of the various factors that significantly affect HIV-related mortality in children. Children with advanced disease stage (WHO-III/IV), TB co-infection, opportunistic infections, immunosuppression with lower CD4 count, poor adherence to medication, and lack of cotrimoxazole use were at greater risk of HIV related mortality in Africa. Moreover, the review also indicted that under 5 years age groups were found to a greater likelihood of TB coinfection, low adherence, and not using cotrimoxazole. Regional differences revealed higher mortality risks in countries such as Zambia and Cameroon due to advanced HIV disease, Nigeria due to severe immunosuppression, and Ethiopia across multiple factors including poor adherence, lack of cotrimoxazole use, and TB co-infection. These findings emphasize the need for age- and region-specific interventions such as early diagnosis in settings with late-stage presentations, improved management of immunosuppression, adherence support and prophylaxis expansion, and targeted TB screening for younger children. Policymakers should prioritize integrated pediatric HIV care strategies, strengthen community-based support systems, and allocate resources to ensure equitable access to these services, particularly in high-burden and under-resourced settings.

## Supplementary Information

Below is the link to the electronic supplementary material.

Supplementary Material 1.

## Data Availability

All the data analyzed during this study are included in this published article.

## References

[1] Enane LA, et al. Traversing the cascade: urgent research priorities for implementing the ‘treat all’strategy for children and adolescents living with HIV in sub-Saharan Africa. J Virus Eradication. 2018;4:40–6. 10.1016/S2055-6640(20)30344-7 PMC6248846 30515313

[2] World health Organization(WHO). HIV/AIDS WHO Africa Report. 2023 [cited 2024 April 22]; Available from: https://www.afro.who.int/health-topics/hivaids#:~:text=Treatment%20and%20care%20in%20children,died%20of%20AIDS%2Drelated%20illnesses

[3] Desmonde S, et al. Time-varying age‐and CD4‐stratified rates of mortality and WHO stage 3 and stage 4 events in children, adolescents and youth 0 to 24 years living with perinatally acquired HIV, before and after antiretroviral therapy initiation in the paediatric IeDEA global cohort consortium. Afr J Reprod Gynaecol Endoscopy. 2020;23(10):e25617. 10.1002/jia2.25617 PMC7545918 33034417

[4] Frigati L, et al. Priorities for decreasing morbidity and mortality in children with advanced HIV disease. Clin Infect Dis. 2018;66(suppl2):S147–51. 29514237 10.1093/cid/ciy013 PMC5850631

[5] World Health Organization. Global HIV Programme: Advanced HIV disease. 2022 [cited 2024 April 28]; Available from: https://www.who.int/teams/global-hiv-hepatitis-and-stis-programmes/hiv/treatment/advanced-hiv-disease#:~:text=WHO%20defines%20advanced%20HIV%20disease,to%20have%20advanced%20HIV%20disease

[6] World health Organization(WHO), Guidelines for managing advanced HIV disease and rapid initiation of antiretroviral therapy. 2017, WHO. p. 56. 29341560

[7] Edessa D, Asefa F, Sheikahmed J. Early mortality among HIV-positive children initiated anti-retroviral therapy in Eastern ethiopia: a retrospective cohort study. Sci Technol Arts Res J. 2015;4(2):157–63.

[8] Mwiru RS, et al. Nutritional status and other baseline predictors of mortality among HIV-infected children initiating antiretroviral therapy in Tanzania. J Int Association Providers AIDS Care (JIAPAC). 2015;14(2):172–9. 10.1177/2325957413500852 PMC4627587 24106055

[9] Nyandiko W et al. Predictors of mortality among children and adolescents living with HIV on antiretroviral therapy in Western Kenya. JAIDS J Acquir Immune Defic Syndr, 2022: pp. 10–1097. 10.1097/QAI.0000000000003361 PMC10896189 38133591

[10] Alebel A, et al. Mortality rate among HIV-positive children on ART in Northwest ethiopia: a historical cohort study. BMC Public Health. 2020;20(1):1–11. 32854692 10.1186/s12889-020-09418-6 PMC7457276

[11] Ben-Farhat J, et al. Mortality and clinical outcomes in children treated with antiretroviral therapy in four African vertical programmes during the first decade of paediatric HIV care, 2001–2010. Volume 22. Tropical Medicine & International Health; 2017. pp. 340–50. 3. 10.1111/tmi.12830 27992677

[12] Davies M-A, et al. Prognosis of children with HIV-1 infection starting antiretroviral therapy in Southern africa: a collaborative analysis of treatment programs. Pediatr Infect Dis J. 2014;33(6):608–16. 24378936 10.1097/INF.0000000000000214 PMC4349941

[13] Mulugeta A, et al. Determinants of survival among HIV positive children on antiretroviral therapy in public hospitals, addis ababa, Ethiopia. Qual Prim Care. 2017;25(4):235–41.

[14] Sidamo N et al. Incidence and predictors of mortality among children on anti-retroviral therapy in public health facilities of Arba minch town, Gamo Gofa zone, Southern ethiopia; retrospective cohort study. Clin Mother Child Health, 2017. 14(3).

[15] Wamalwa DC, et al. Predictors of mortality in HIV-1 infected children on antiretroviral therapy in kenya: a prospective cohort. BMC Pediatr. 2010;10(1):33. 20482796 10.1186/1471-2431-10-33 PMC2887829

[16] Zanoni BC, et al. Risk factors associated with increased mortality among HIV infected children initiating antiretroviral therapy (ART) in South Africa. PLoS ONE. 2011;6(7):e22706. 21829487 10.1371/journal.pone.0022706 PMC3146475

[17] Ahmed I, Lemma S. Mortality among pediatric patients on HIV treatment in sub-Saharan African countries: a systematic review and meta-analysis. BMC Public Health. 2019;19(1):149. 30717720 10.1186/s12889-019-6482-1 PMC6360742

[18] Page MJ, et al. The PRISMA 2020 statement: an updated guideline for reporting systematic reviews. Int J Surg. 2021;88:105906. 33789826 10.1016/j.ijsu.2021.105906

[19] Herzog R, et al. Newcastle-Ottawa scale adapted for cross-sectional studies. BMC Public Health. 2013;13:154. 23421987 10.1186/1471-2458-13-154 PMC3602084

[20] Biyazin Y, et al. Survival and predictors of mortality among HIV-positive children on antiretroviral therapy in public hospitals. J Pharm Policy Pract. 2022;15(1):48. 35978382 10.1186/s40545-022-00448-6 PMC9382771

[21] Gebremedhin A, et al. Predictors of mortality among HIV infected children on anti-retroviral therapy in Mekelle hospital, Northern ethiopia: a retrospective cohort study. BMC Public Health. 2013;13:1–6. 24517533 10.1186/1471-2458-13-1047 PMC4028824

[22] Koye DN, Ayele TA, Zeleke BM. Predictors of mortality among children on antiretroviral therapy at a referral hospital, Northwest ethiopia: a retrospective follow up study. BMC Pediatr. 2012;12(1):1–7. 23043325 10.1186/1471-2431-12-161 PMC3478986

[23] Tagesse N, Abebe W. Predictors of mortality in children and adolescents living with HIV on antiretroviral therapy, ethiopia: A retrospective cohort study. Ethiop J Pediatr Child Health, 2020. 15(2).

[24] Alemu GG, et al. Survival time and predictors of death among HIV infected under five children after initiation of anti-retroviral therapy in West Amhara referral hospitals, Northwest Ethiopia. BMC Pediatr. 2022;22(1):670. 36411424 10.1186/s12887-022-03693-5 PMC9677693

[25] Bitew S, Mekonen A, Assegid M. Predictors on mortality of human immunodeficiency virus infected children after initiation of antiretroviral treatment in Wolaita zone health facilities, ethiopia: retrospective cohort study. J AIDS HIV Res. 2017;9(4):89–97.

[26] Higgins JP, Thompson SG. Quantifying heterogeneity in a meta-analysis. Stat Med. 2002;21(11):1539–58. 12111919 10.1002/sim.1186

[27] Cochran WG. The combination of estimates from different experiments. Biometrics. 1954;10(1):101–29.

[28] Moges S, et al. The impact of undernutrition and anemia on HIV-Related mortality among children on ART in Sub-Saharan africa: A systematic review and Meta-Analysis. J Epidemiol Global Health. 2024;14(4):1453–63. 10.1007/s44197-024-00321-6 PMC11652469 39541033

[29] Adem AK, Alem D, Girmatsion F. Factors affecting survival of HIV positive children taking antiretroviral therapy at Adama referral hospital and medical college, Ethiopia. J AIDS Clin Res, 2014. 5(3).

[30] Belachew A. *Predictors of Survival Time among Children On Antiretroviral Therapy In Dilchora Referral Hospital, Dire Dawa, East Ethiopia.* 2019.

[31] Chekole B, et al. Survival status and predictors of mortality among HIV-positive children initiated antiretroviral therapy in Bahir Dar town public health facilities Amhara region, ethiopia, 2020. SAGE Open Med. 2022;10:p20503121211069477. 10.1177/20503121211069477 PMC8793112 35096391

[32] Ebissa G, Deyessa N, Biadgilign S. Predictors of early mortality in a cohort of HIV-infected children receiving high active antiretroviral treatment in public hospitals in Ethiopia. AIDS Care. 2015;27(6):723–30. 25599414 10.1080/09540121.2014.997180

[33] Marie BT, Argaw WS, Bazezew BY. Time to death among HIV-infected under-five children after initiation of anti-retroviral therapy and its predictors in Oromiya Liyu zone, Amhara region, ethiopia: a retrospective cohort study. BMC Pediatr. 2022;22:1–9. 35538445 10.1186/s12887-022-03295-1 PMC9088057

[34] Mekonnen E, et al. Time to death and its predictors among under-five children on antiretroviral treatment in public hospitals of addis ababa, addis ababa, ethiopia, a retrospective follow up study. PLoS ONE. 2023;18(7):e0288475. 37471340 10.1371/journal.pone.0288475 PMC10358914

[35] Melaku Z, et al. Outcomes among HIV-infected children initiating HIV care and antiretroviral treatment in Ethiopia. Tropical Med Int Health. 2017;22(4):474–84. 10.1111/tmi.12834 PMC11541046 28066962

[36] Molla M, et al. Effects of undernutrition and predictors on the survival status of HIV-Positive children after started antiretroviral therapy (ART) in Northwest Ethiopia. Int J Pediatr. 2022;2022(1):1046220. 35222650 10.1155/2022/1046220 PMC8872677

[37] Oumer A, Kubsa ME, Mekonnen BA. Malnutrition as predictor of survival from anti-retroviral treatment among children living with HIV/AIDS in Southwest ethiopia: survival analysis. BMC Pediatr. 2019;19:1–10. 31801487 10.1186/s12887-019-1823-x PMC6892183

[38] Arage G, et al. Survival rate of HIV-infected children after initiation of the antiretroviral therapy and its predictors in ethiopia: a facility-based retrospective cohort. SAGE Open Med. 2019;7:2050312119838957. 30937168 10.1177/2050312119838957 PMC6434434

[39] Brophy JC, et al. Survival outcomes in a pediatric antiretroviral treatment cohort in Southern Malawi. PLoS ONE. 2016;11(11):e0165772. 27812166 10.1371/journal.pone.0165772 PMC5094712

[40] Fetzer BC, et al. Predictors for mortality and loss to follow-up among children receiving anti-retroviral therapy in lilongwe, Malawi. Tropical Med Int Health. 2009;14(8):862–9. 10.1111/j.1365-3156.2009.02315.x PMC2892779 19563431

[41] Munthali T, et al. Survival of children living with HIV on Art in zambia: A 13-Years retrospective cohort analysis. Front Public Health. 2020;8:96. 32296674 10.3389/fpubh.2020.00096 PMC7138171

[42] Mutanga JN, et al. Long-term survival outcomes of HIV infected children receiving antiretroviral therapy: an observational study from Zambia (2003–2015). BMC Public Health. 2019;19:1–12. 30691416 10.1186/s12889-019-6444-7 PMC6348639

[43] Abrams EJ, et al. Despite access to antiretrovirals for prevention and treatment, high rates of mortality persist among HIV-infected infants and young children. Pediatr Infect Dis J. 2017;36(6):595–601. 28027287 10.1097/INF.0000000000001507 PMC5432395

[44] Kabue MM, et al. Mortality and clinical outcomes in HIV-infected children on antiretroviral therapy in malawi, lesotho, and Swaziland. Pediatrics. 2012;130(3):e591–9. 22891234 10.1542/peds.2011-1187 PMC3962849

[45] Auld AF, et al. Temporal trends in mortality and loss to follow-up among children enrolled in Cote d’ivoire’s National antiretroviral therapy program. Pediatr Infect Dis J. 2014;33(11):1134–40. 25093975 10.1097/INF.0000000000000457 PMC11440633

[46] Nlend AEN, Loussikila AB. Predictors of mortality among HIV-infected children receiving highly active antiretroviral therapy. Med Et Maladies Infectieuses. 2017;47(1):32–7. 10.1016/j.medmal.2016.07.003 27609595

[47] Nugent J, et al. Predicting mortality in HIV-infected children initiating highly active antiretroviral therapy in a resource-deprived setting. Pediatr Infect Dis J. 2014;33(11):1148–55. 24945879 10.1097/INF.0000000000000454

[48] Anigilaje EA, Aderibigbe SA. Mortality in a cohort of HIV-Infected children: A 12‐Month outcome of antiretroviral therapy in makurdi, Nigeria. Volume 2018. Advances in medicine; 2018. p. 6409134. 1. 10.1155/2018/6409134 PMC6029505 30018988

[49] McHugh G et al. Clinical outcomes in children and adolescents initiating antiretroviral therapy in decentralized healthcare settings in Zimbabwe. Afr J Reprod Gynaecol Endoscopy, 2017. 20(1). 10.7448/IAS.20.1.21843 PMC5719665 28872269

[50] Andargie AA, Asmleash Y. Survival time of human immunodeficiency virus (HIV) infected children under 15 years of age after initiation of antiretroviral therapy in the university of Gondar comprehensive specialized hospital, Ethiopia. J AIDS HIV Res. 2018;10(4):49–55.

[51] Zhang H, et al. Long-term effect of antiretroviral therapy on mortality among HIV-positive children and adolescents in China. Heliyon. 2024;10(7):e27961. 38596025 10.1016/j.heliyon.2024.e27961 PMC11002537

[52] Woldegeorgis BZ, et al. Mortality and its predictors among human immunodeficiency virus-infected children younger than 15 years receiving antiretroviral therapy in ethiopia: a systematic review and meta-analysis. BMC Infect Dis. 2024;24(1):471. 38702591 10.1186/s12879-024-09366-1 PMC11069260

[53] Mollel EW, et al. Effect of tuberculosis infection on mortality of HIV-infected patients in Northern Tanzania. Trop Med Health. 2020;48:1–10. 32355448 10.1186/s41182-020-00212-z PMC7184680

[54] Hamada Y, et al. HIV-associated tuberculosis. Int J STD AIDS. 2021;32(9):780–90. 33612015 10.1177/0956462421992257 PMC8236666

[55] Kay A, et al. Predicting mortality within 1 year of ART initiation in children and adolescents living with HIV in sub-Saharan africa: a retrospective observational cohort study. Lancet Global Health. 2024;12(6):e929–37. 38762295 10.1016/S2214-109X(24)00091-3 PMC11149103

[56] Holmberg PJ, Temesgen Z, Banerjee R. Tuberculosis in children. Pediatr Rev. 2019;40(4):168–78. 30936398 10.1542/pir.2018-0093

[57] Lakoh S, et al. Causes of hospitalization and predictors of HIV-associated mortality at the main referral hospital in Sierra leone: a prospective study. BMC Public Health. 2019;19:1–9. 31638941 10.1186/s12889-019-7614-3 PMC6805411

[58] Mangal TD, et al. Determinants of survival of people living with HIV/AIDS on antiretroviral therapy in Brazil 2006–2015. BMC Infect Dis. 2019;19:1–9. 30819120 10.1186/s12879-019-3844-3 PMC6396460

[59] Lumbiganon P, et al. Survival of HIV-infected children: a cohort study from the Asia-Pacific region. J Acquir Immune Defic Syndr. 2011;56(4):365–71. 21160429 10.1097/QAI.0b013e318207a55b PMC3072816

[60] Judd A, et al. Long-term trends in mortality and AIDS-defining events after combination ART initiation among children and adolescents with perinatal HIV infection in 17 middle- and high-income countries in Europe and thailand: A cohort study. PLoS Med. 2018;15(1):e1002491. 29381702 10.1371/journal.pmed.1002491 PMC5790238

[61] The European P, H.I.V.C.C.S.G.i E, Paediatric. Prevalence and clinical outcomes of poor immune response despite virologically suppressive antiretroviral therapy among children and adolescents with human immunodeficiency virus in Europe and thailand: cohort study. Clin Infect Dis. 2020;70(3):404–15. 30919882 10.1093/cid/ciz253

[62] van Wyk BE, Davids L-AC. Challenges to HIV treatment adherence amongst adolescents in a low socio-economic setting in cape town. South Afr J HIV Med. 2019;20(1):1–7. 10.4102/sajhivmed.v20i1.1002 PMC6852420 31745433

[63] Mandalakas AM, et al. Tuberculosis among children and adolescents at HIV treatment centers in Sub-Saharan Africa. Emerg Infect Dis. 2020;26(12):2933–43. 33219815 10.3201/eid2612.202245 PMC7706926

[64] Mathur S, et al. Estimating the impact of alternative programmatic Cotrimoxazole strategies on mortality among children born to mothers with HIV: A modelling study. PLoS Med. 2024;21(2):e1004334. 38377150 10.1371/journal.pmed.1004334 PMC10914273

[65] Boettiger DC, et al. Temporal trends in co-trimoxazole use among children on antiretroviral therapy and the impact of co-trimoxazole on mortality rates in children without severe immunodeficiency. J Pediatr Infect Dis Soc. 2019;8(5):450–60. 10.1093/jpids/piy087 PMC6831936 30215763

[66] Chintu C, et al. Co-trimoxazole as prophylaxis against opportunistic infections in HIV-infected Zambian children (CHAP): a double-blind randomised placebo-controlled trial. Lancet. 2004;364(9448):1865–71. 15555666 10.1016/S0140-6736(04)17442-4

[67] Graham SM. Prophylaxis against Pneumocystis carinii pneumonia for HIV-exposed infants in Africa. Lancet. 2002;360(9349):1966–8. 12493279 10.1016/S0140-6736(02)11921-0

[68] Zhu JH, et al. Effects of Cotrimoxazole prophylaxis initiation and discontinuation on mortality and attrition rates among HIV patients who initiate ART in Southwest china: an observational cohort study. Biomed Environ Sci. 2021;34(8):646–9. 34474726 10.3967/bes2021.090

[69] Kifude CM, et al. Initiation of anti-retroviral/Trimethoprim-Sulfamethoxazole therapy in a longitudinal cohort of HIV-1 positive individuals in Western Kenya rapidly decreases asymptomatic malarial parasitemia. Front Cell Infect Microbiol. 2022;12:1025944. 36506016 10.3389/fcimb.2022.1025944 PMC9729353

