# Supplementary material for: Determinants of HIV related mortality in African children on antiretroviral therapy: clinical and immunological insights from a systematic review and meta-analysis
Source: AIDS Res Ther. 2025 Oct 8;22:102. doi: 10.1186/s12981-025-00784-y (PMC12509419; doi:10.1186/s12981-025-00784-y)
Supplement: Supplementary file 1 — Supplementary Material 1. [file 12981_2025_784_MOESM1_ESM.docx]

Supplementary file : Assessment of risk of bias and quality of studies

| **Study** | **Selection [max. of 4 stars]** | **Comparability [max. of 2 stars]** | **Outcome [max. of 3 stars]** | **Total score** | **Study quality** |
| --- | --- | --- | --- | --- | --- |
| Ebissa et al., 2015 [31] | 3 | 1 | 2 | 6 | Moderate |
| Koye et al., 2012 [22] | 3 | 2 | 3 | 8 | High |
| Munthali et al., 2020 [40] | 4 | 2 | 3 | 9 | High |
| Chekole et al., 2022 [30] | 3 | 1 | 2 | 6 | Moderate |
| Anigilaje & Aderibigbe, 2018 [47] | 3 | 2 | 2 | 7 | High |
| Bitew et al., 2017 [25] | 3 | 2 | 2 | 7 | High |
| Mekonnen et al., 2023 [33] | 3 | 2 | 3 | 8 | High |
| Alebel et al., 2020 [12] | 3 | 2 | 3 | 8 | High |
| Tagesse & Abebe, 2020 [23] | 3 | 2 | 2 | 7 | High |
| Edessa et al., 2015 [9] | 3 | 2 | 2 | 7 | High |
| Adem et al., 2014 [28] | 3 | 2 | 2 | 7 | High |
| Abrams et al., 2017 [42] | 3 | 2 | 3 | 8 | High |
| Mulugeta et al., 2017[15] | 3 | 1 | 2 | 6 | Moderate |
| Auld et al., 2014 [44] | 3 | 2 | 3 | 8 | High |
| Ben‐Farhat et al., 2017 [13] | 3 | 2 | 3 | 8 | High |
| Brophy et al., 2016 [38] | 3 | 2 | 3 | 8 | High |
| Sidamo et al., 2017 [16] | 3 | 2 | 2 | 7 | High |
| Marie et al., 2022 [32] | 3 | 2 | 2 | 7 | High |
| Davies et al., 2014 [14] | 3 | 2 | 3 | 8 | High |
| McHugh et al., 2017 [48] | 3 | 2 | 2 | 7 | High |
| Biyazin et al., 2022 [20] | 3 | 2 | 2 | 7 | High |
| Nlend & Loussikila, 2017[45] | 3 | 2 | 2 | 7 | High |
| Nyandiko et al., 2022 [11] | 3 | 2 | 3 | 8 | High |
| Gebremedhin et al., 2013 [21] | 3 | 2 | 2 | 7 | High |
| Arage et al., 2019 [37] | 3 | 2 | 2 | 7 | High |
| Mwiru et al., 2015 [10] | 3 | 2 | 2 | 7 | High |
| Zanoni et al., 2011 [18] | 3 | 2 | 3 | 8 | High |
| Andargie & Asmleash, 2018 [49] | 3 | 2 | 2 | 7 | High |
| Melaku et al., 2017 [34] | 3 | 2 | 2 | 7 | High |
| Kabue et al., 2012 [43] | 3 | 2 | 3 | 8 | High |
| Oumer et al., 2019 [36] | 3 | 2 | 2 | 7 | High |
| Molla et al., 2022 [35] | 3 | 2 | 2 | 7 | High |
| Mutanga et al., 2019 [41] | 3 | 1 | 3 | 7 | High |
| Nugent et al., 2014 [46] | 3 | 2 | 3 | 8 | High |
| Alemu et al., 2022 [24] | 3 | 2 | 2 | 7 | High |
| Fetzer et al., 2009 [39] | 3 | 2 | 2 | 7 | High |
